# Proteomic analysis reveals candidate molecules to mediate cortical pathology and identify possible biomarkers in an animal model of multiple sclerosis

**DOI:** 10.3389/fimmu.2025.1505459

**Published:** 2025-02-13

**Authors:** Berenice Anabel Silva, María Celeste Leal, María Isabel Farias, Agustín Nava, Daniela Inés Galván, Elmer Fernandez, Fernando Juan Pitossi, Carina Cintia Ferrari

**Affiliations:** ^1^ Fundación Instituto Leloir (FIL), Instituto de Investigaciones Bioquímicas de Buenos Aires (IIBBA), Consejo Nacional de Investigaciones Científicas y Técnicas (CONICET), Buenos Aires, Argentina; ^2^ Fundación Huésped, Buenos Aires, Argentina; ^3^ ScireLab, Fundación para el Progreso de la Medicina, CONICET, Córdoba, Argentina; ^4^ Facultad de Ciencias Exactas, Físicas y Naturales (FCEFyN), Universidad Nacional de Córdoba (UNC), Córdoba, Argentina

**Keywords:** cortex, cerebrospinal fluid, interleukin-1β, demyelination, neurodegeneration, neuroinflammation, orosomucoid-1, S100A8

## Abstract

**Introduction:**

Multiple Sclerosis (MS) is a complex neurodegenerative disease marked by recurring inflammatory episodes, demyelination, axonal damage, and subsequent loss of function. MS presents a wide range of clinical courses, with the progressive forms leading to irreversible neurological disability. Cortical demyelinating lesions are central to the pathology of these progressive forms, gaining critical importance in recent decades due to their strong correlation with physical disability and cognitive decline. Despite this, the underlying mechanisms driving cortical lesion formation remain poorly understood, and no specific treatments are currently available. A significant challenge lies in the lack of animal models that accurately mirror the key characteristics of these lesions.

**Methods:**

We developed a focal cortical animal model that replicates many features of cortical lesions, including cognitive impairment. This study focuses on conducting proteomic analyses of both the cortical lesions and cerebrospinal fluid (CSF) from these animals, aiming to identify key proteins and biomarkers that could be validated in MS patients.

**Results:**

Proteomic differences between frontal cortex tissue and CSF were observed when comparing experimental animals with controls. Among the identified proteins, some have been previously described in MS patients and animal models, while others represent novel discoveries. Notably, we identified two proteins, S100A8 and orosomucoid-1, that were highly expressed in both regions.

**Conclusions:**

These findings suggest that the prognostic molecules identified in this model could facilitate the discovery of new biomarkers or key molecules relevant to MS, particularly in the cortical lesion that mainly characterized the progressive forms of the disease.

## 1 Introduction

Multiple sclerosis (MS) is a neurodegenerative disease characterized by recurrent inflammatory events, demyelination, and axonal damage, along with loss of function (1). Furthermore, MS is a very heterogeneous disease that displays different clinical courses, including episodes of relapse followed by remission of symptoms [‘‘relapsing-remitting MS” (RRMS)], which can progress to a secondary progressive form (SPMS). In addition, patients may experience persistent progression from the onset of the disease [‘‘primary progressive MS” (PPMS)] (2). The progressive forms of MS (SPMS and PPMS) lead to patients acquiring a major irreversible neurological disability that severely affects their quality of life. Currently, cortical demyelination is considered a key feature in PPMS and SPMS patients and is less frequent in RRMS (3). These cortical lesions have clinical relevance because they show a stronger correlation with physical disability and cognitive impairment in several studies, even more frequently than those described for white matter lesions (4–7). Notably, it is noteworthy that the presence of cortical lesions at diagnosis has recently been shown to predict long-term cognitive decline in MS in a 20-year study (8). The pathogenesis of cortical lesions is still unknown. However, neurodegeneration and glial activation are predominant in these lesions. Therefore, the cortical microenvironment may influence the degree of inflammation, tissue damage, and lesion repair (9). These plaques of primary demyelination in the cortex represent a -specific key feature of MS, and they are not seen in other chronic neuroinflammatory conditions (10). To date, there are no available treatments for cortical lesions, mostly because research has been hampered by the availability of few animal models that reflect these characteristics (11). The majority of these few models have been developed based on experimental autoimmune encephalomyelitis (EAE) along with focal injection of cytokines. These lesions are characterized by demyelination, neuroinflammation, glial activation, and neuronal degeneration (11–15). These features, along with the chronicity of the lesions, vary depending on the animal model. Proteomic studies have not been performed in any of these animal models.

We have developed a focal cortical animal model triggered by the chronic expression of a prototypical pro-inflammatory cytokine, IL-1β, in the cortex and further peripheral inflammatory stimulation (16). In our model, the cortical lesions are characterized by demyelination, neurodegeneration, microglial/macrophage activation, and associated meningeal inflammation, lasting for at least 50 days. These pathological features correlate with impaired cognitive performance and anxiety-like symptoms in the animals. Interestingly, the meninges contain follicle-like structures very similar to those mainly observed in SPMS patients (16). This model represents one of five models that resemble MS cortical lesions, but it is the only one that exhibits a chronicity similar to that found in MS patients (12, 15, 17).

Additionally, there are current studies of the cerebrospinal fluid (CSF) and cortical tissue proteome in some animal models of MS, including EAE and cuprizone in rats. In the CSF of EAE, the authors found several important proteins, among them afamin and complement C3, which cause an increase in blood–brain barrier (BBB) permeability (18). The Frontal cortex proteomics of cuprizone and EAE mice also revealed several differential proteins such as Legumain and C1Q in cuprizone and hemopexin in EAE (19). Legumain was also found to be significantly higher in active and chronic lesions of postmortem MS brain tissue. Recently, the dorsal cortex and the spinal cord of two MS models (EAE and cuprizone) have demonstrated that orosomucoid-1 (Orm1) consistently exhibited alterations in both models and regions (20).

Here, we investigated the proteome of both cortical lesions and CSF in a novel cortical MS animal model (16). Our hypothesis posits that cortical lesions are mediated by key molecules contributing to inflammation, neurodegeneration, and behavioral symptoms. The objective of this study is to identify novel molecular pathways activated in the cortex and the CSF that may influence the formation and progression of cortical MS lesions as well as early specific indicators in the development of these lesions. Proteomic analysis of cortical lesions in this model aims to identify key proteins involved in the pathophysiology of cortical damage that could be validated in MS patients.

## 2 Materials and methods

### 2.1 Animals and surgeries

For all experiments, adult male Wistar rats were used (original colony obtained from the Jackson Laboratory, Bar Harbor, Maine, USA) and maintained for several generations at the Animal Facility of the Leloir Institute Foundation. The rats were 8–10 weeks old. The animals were kept in the vivarium at a constant temperature of 22 ± 2°C, under a light:dark cycle of 12:12 h, and with food and water available *ad libitum*. All procedures were performed following the regulations of the National Institutes of Health (NIH) of the United States and the internal regulations of the Leloir Institute Foundation, with the approval of the Institutional Commission for the Care and Use of Laboratory Animals of the Institute (CICUAL - FIL).

Intracerebral stereotaxic injections were performed in the morning to avoid the possible effects of circadian variations in cytokine expression on inflammatory responses. Animals were anesthetized with ketamine hydrochloride (80 mg/kg) and xylazine (8 mg/kg), and injected into the left prefrontal cortex (bregma, +1.6 mm; lateral, +2.5 mm; ventral, −1.6 mm) (21) with adenovirus expressing IL-1β (AdIL-1β) or adenovirus expressing β-galactosidase (Adβ-gal) as control. They were made with a drawn glass capillary (approximate diameter of 50 nm) in order to minimize nerve tissue damage due to surgery *per se*. The vectors were diluted in sterile 10 mM Tris-Cl buffer (pH 7.8) to a final concentration of 1 × 10^6^ infectious particles/μL. A final volume of 1 μL was injected. The injections were performed over a period of 5 min: in the first 4 min, the vector was administered (0.25 μL/min), and then the capillary was held in place for an additional minute in order to avoid reflux of the solution. Finally, the wound was cleaned and closed by suturing. The animals were kept under a lamp that provided heat until they recovered from anesthesia.

### 2.2 Peripheral booster stimuli

**Figure 1:**
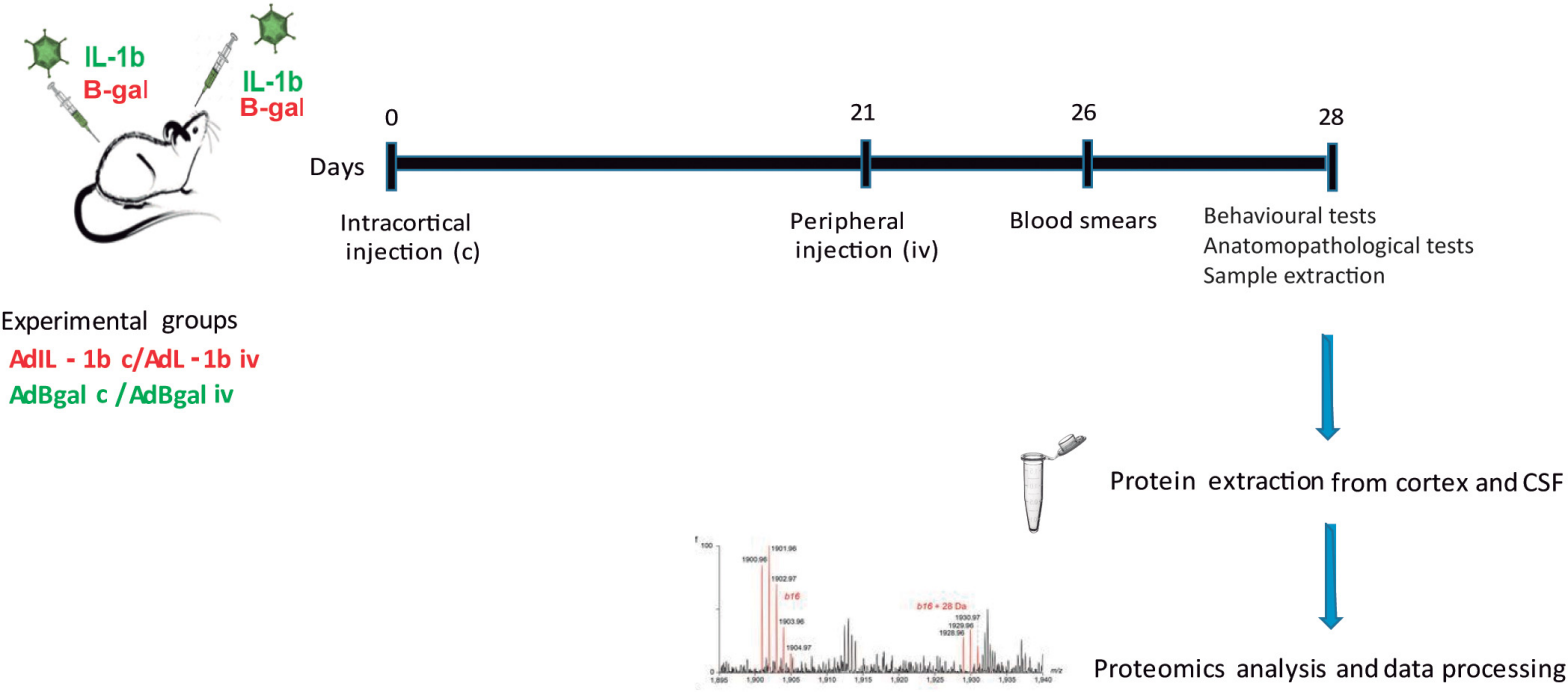
Experimental design. Animals received a central inflammatory stimulus on day 0 of AdIL-1β c or Adβ-gal c, and a peripheral injection of AdIL-1β p or Adβ-gal p after 21 days. The studies were performed at 7 days after the peripheral stimulation. Therefore, we have two experimental groups [AdIL-1β c/AdIL-1β p (experimental animals) and Adβ-gal c/Adβ-gal p (control animals)]. *n* = 5 per group.

A total of 21 days after the central injection in the cortex, the peripheral stimulus was applied. AdIL-1β was used as a pro-inflammatory stimulus, applied intravenously (iv) from the tail vein of the animal. Adβ-gal was used as a control (**Figure 1**). The animals were anesthetized for a short period of time by inhalation of Isoflurane; once asleep, they were placed under light and the lateral tail vein was located and the injection was performed with a 27G needle: first, they were passed 300 μL of the adenovector at a concentration of 1.4 × 10^9^ infectious particles/μL, diluted in sterile 10 mM Tris-Cl buffer (pH 7.8); second, 300 μL of the sterile physiological solution was passed. Once the animals recovered from anesthesia, they were returned to their respective cages. The effectiveness of peripheral stimulation was verified by counting the peripheral leukocytes in the blood of the animals injected with both the AdIL-1β and the control adenovector. To do this, a drop of blood was taken from the tail of the rats and smeared on a glass slide. Once dry, the samples were stained with the May-Grünwald/Giemsa (MGG) technique, which allows qualitative and quantitative differentiation of the main blood components. The sample from which the blood smears were performed was taken 5 days after the IV injection since previous laboratory work had shown that this was the day when the peak of peripheral AdIL-1β effect was observed. In these experiments, to test functionality, it is expected for the peripheral AdIL-1β-injected animals to exhibit a significant increase in the number of neutrophils, together with a consequent decrease in the lymphocyte population, which is significantly different from the effects observed in the peripheral Adβ-gal-injected animals. As previously described, we evaluated the functionality of the animal model through various behavioral tests, including the open-field test and anxiety-like assessments, along with histological analyses (**Supplementary Figures S1**, **S2**). Additionally, we examined the anatomopathological features of the animals, similar to our previous analyses conducted on control animals for the proteomic experiments (16). We performed Cresyl Violet staining to assess general nervous tissue integrity and inflammation, alongside immunohistochemistry to evaluate demyelination, glial activation, and neurodegeneration. The results were consistent with those reported in our manuscript detailing the model, confirming cortical demyelination, neurodegeneration, and glial activation (16).

In this work, we studied two groups: cortical IL-1β/peripheral IL-1β (IL-1β c/IL-1β iv) (*n* = 5) and cortical β-gal/peripheral β-gal (*n* = 5) (β-gal c/β-gal iv) as controls in order to obtain the greatest differential protein expression in each condition at 21 + 7 dpi, 7 days after intravenous injection of IL-1β or β-gal (**Figure 1**).

### 2.3 Cortical lesion sampling

On day 28, rat brains were excised postmortem, and the frontal cortex of the left hemisphere anterior to bregma, +1.6 mm, was collected by dissection and immediately stored at −80°C until further processing for proteomic analysis.

### 2.4 CSF sampling

On day 28, animals were anesthetized as previously described in the Animals and surgeries section; and the head was fixed in a stereotaxic apparatus. A skin incision was made, followed by a horizontal incision in the dorsal muscle. An insulin needle (27G × ½") was inserted into the cisterna magna, allowing us to obtain a maximum of 100 μL per animal. The samples were maintained on ice until centrifugation at 2,000*g* for 10 min at 4°C. The supernatant was aliquoted into 50 μL tubes and stored at −80°C until further processing for proteomics.

### 2.5 Proteomic analysis of cortical lesions

#### 2.5.1 Preparation of cortical lesion samples

Individual cortical tissues (*n* = 5) were homogenized in RIPA-modified buffer (50 nM Tris-HCl, pH 7.5, 150 mM NaCl, 1 mM EDTA, 1% NP-40, and 0.1% sodium deoxycholate) with the addition of 5 mM b-glycerophosphate, 10 mM sodium fluoride, 10 mM sodium orthovanadate, and protease inhibitors from the Complete Protease Inhibitor Cocktail (Roche, Basel, Switzerland). Samples were sonicated for 5 min with 30 on/off cycles, kept on ice, and centrifuged for 10 min at 10,000 rpm at 4°C. Proteins from the supernatant were precipitated overnight at −20°C by adding a volume of ice-acetone in sixfold excess. The acetone-precipitated proteins were solubilized in a denaturation buffer (6 M urea and 200 mM ammonium bicarbonate in water). The final protein content was reduced and quantified using the Pierce BCA assay (Thermo Fisher, CA, USA). Proteins were reduced with dithiothreitol (DTT 1, 10 mM, 37°C, 60 min) and alkylated with iodoacetamide (IAM, 20 mM, 23°C, 30 min). Then, samples were diluted with 200 mM ammonium bicarbonate up to 2 M urea, digested overnight with Lys-C at 37°C, and then diluted again twofold and digested with trypsin at 37°C. Peptides were desalted using a C18 MicroSpin 300A silica column. (The Nest Group Inc, Ipswich, USA), evaporated to dryness using a SpeedVac and dissolved in 30 µL of 0.1% formic acid in water. The peptides obtained from the cortical lesions were purified using Preomics iST- BCT Sample Preparation kit 96x (Preomics, Martinsried, Germany). Then, 10 samples (5 cortical IL-1β/peripheral IL-1β and 5 cortical β-gal/peripheral β-gal) were sent to the Proteomics Unit of the Pompeu Fabra University (CRG-UPF), Spain in order to analyze the differential expression of lesion proteins.

#### 2.5.2 Preparation of CSF samples

50µl of CSF (*n*=5) were purified using Proteomics IST-BCT Sample Preparation Kit for fluids (Preomics, Martinsried, Germany). The CSF was processed according to the manufacturer’s instructions. Five samples of each treatment were sent to the Proteomics Unit of the Natural Science Faculty of the University of Buenos Aires in order to analyze the differential expression of CSF proteins.

### 2.6 Chromatographic and mass spectrometric analysis

#### 2.6.1 Analysis of cortical lesions

Samples were analyzed using an Orbitrap Eclipse mass spectrometer (Thermo Fisher Scientific, San Jose, CA, USA) coupled to an EASY-nLC 1200 [Thermo Fisher Scientific (Proxeon), Odense, Denmark]. Peptides were loaded directly onto the analytical column and were separated by reversed-phase chromatography using a 50-cm column with an inner diameter of 75 μm, packed with 2-μm C18 particles.

Chromatographic gradients were started at 95% buffer A and 5% buffer B at a flow rate of 300 nL/min and gradually increased to 25% buffer B and 75% buffer A in 79 min and then to 40% buffer B and 60% buffer A in 11 min. After each analysis, the column was washed for 10 min with 100% buffer B. Buffer A: 0.1% formic acid in water. Buffer B: 0.1% formic acid in 80% acetonitrile. The mass spectrometer was operated in positive ionization mode with a nanospray voltage set at 2.4 kV and a source temperature of 305°C. The acquisition was performed in data-dependent acquisition (DDA) mode, and full MS scans with 1-µm scans at a resolution of 120,000 were used over a mass range of *m*/*z* 350–1400 with detection in the Orbitrap mass analyzer. Auto gain control (AGC) was set to “standard” and injection time was set to “auto”. In each cycle of DDA analysis, following each survey scan, the most intense ions above a threshold ion count of 10,000 were selected for fragmentation. The number of selected precursor ions for fragmentation was determined by the “Top Speed” acquisition algorithm and a dynamic exclusion time of 60 s. Fragment ion spectra were produced via high-energy collision dissociation (HCD) at a normalized collision energy of 28%, and they were acquired in the ion trap mass analyzer. The AGC was set to 2E4, and an isolation window of 0.7 *m*/*z* and a maximum injection time of 12 ms were used. Digested bovine serum albumin (New England Biolabs, Ipswich, Massachusetts) was analyzed between each sample to avoid sample carryover and to ensure the stability of the instrument, and QCloud 1 was used to control the longitudinal performance of the instrument during the project.

#### 2.6.2 Analysis of CSF

For chromatographic gradients, the samples were treated by reducing them with 20 mM DTT for 45 min at 56°C and alkylating them with 50 mM iodoacetamide for 45 min in the dark. Subsequently, they were digested with trypsin, which cleaves the peptide bonds of Arg and Lys on the C-terminal side, throughout the night. Peptide extraction was carried out using acetonitrile. The samples were lyophilized via SpeedVac and then reconstituted in 30 μL of 0.1% trifluoroacetic acid. Desalting was performed using the Zip Tip C18 column from Merck^®^. Analysis was conducted by nanoHPLC coupled to a mass spectrometer with Orbitrap technology, allowing for the separation of peptides obtained from trypsin digestion of the sample and subsequent identification. Sample ionization was achieved through electrospray.

The Mass Spectrometer was a Q-Exactive (Thermo Scientific, CA, USA), which has a High Collision Dissociation (HCD) cell and an Orbitrap analyzer. The configuration of the equipment enables peptide identification concurrently with chromatographic separation, yielding full MS and MSMS. A method was employed to maximize the number of measurement cycles per unit of time. The cycle duration depends on the chosen resolution. In each cycle, the equipment performs a Full MS and subsequently conducts MSMS on the 12 peaks with the best signal-to-noise ratio in that cycle, employing a dynamic exclusion range to reduce the frequency of peptide fragmentation during its chromatographic elution. The spectra were analyzed with the Proteome Discoverer program (Perseus).

### 2.7 Proteomic data analysis

#### 2.7.1 Cortex

The acquired spectra were analyzed using the Proteome Discoverer software suite (v2.0, Thermo Fisher Scientific) and the Mascot search engine (v2.6, Matrix Science 2). The data were searched against a Uniprot_Rat database (as of March 2023, 47,943 entries) plus a list of the three most common contaminants and all the corresponding decoy entries. For peptide identification, a precursor ion mass tolerance of 7 parts per million (ppm) was used for the MS1 level, trypsin was chosen as the enzyme, and up to three missed cleavages were allowed. The fragment ion mass tolerance was set to 0.5 Da for MS2 spectra. Oxidation of methionine and N-terminal protein acetylation were used as variable modifications, whereas carbamidomethylation on cysteines was set as a fixed modification. The false discovery rate (FDR) for peptide identification was set to a maximum of 5%. Peptide quantification data were retrieved from the “Precursor ions quantifier” node of Proteome Discoverer (v2.5) using a 2 ppm mass tolerance for the peptide extracted ion current (XIC). The obtained values were used to calculate protein fold changes and their corresponding *p*-values and adjusted p-values. The raw proteomic data have been deposited in the PRIDE 4 repository under the dataset identifier PXD050417.7.2. CSF. The digests were analyzed by nanoLC-MS/MS in a Thermo Scientific Q-Exactive Mass Spectrometer coupled to a nanoHPLC EASY-nLC 1000 (Thermo Scientific). For the LC-MS/MS analysis, approximately 1 μg of peptide was loaded onto the column and eluted for 120 min using a reverse-phase column (C18, 2 µm, 100 A, 50 µm × 150 mm) Easy-Spray Column PepMap RSLC (P/N ES801), which is suitable for separating protein complexes with a high degree of resolution. The flow rate used for the nano column was 300 nL min^−1^ and the solvent ranged from 7% B (5 min) to 35% (120 min). Solvent A was 0.1% formic acid in water, whereas solvent B was 0.1% formic acid in acetonitrile. The injection volume was 2 µL. The MS equipment has an HCD cell for fragmentation and an Orbitrap analyzer (Thermo Scientific, Q-Exactive). A voltage of 3,5 kV was used for ElectroSpray Ionization (Thermo Scientific, EASY-SPRAY, CA, USA)). XCalibur 3.0.63 (Thermo Scientific, CA, USA) software was used for data acquisition and equipment configuration, allowing peptide identification concurrently with chromatographic separation. Full-scan mass spectra were acquired on the Orbitrap analyzer. The scanned mass range was 400–1,800 *m*/*z* at a resolution of 70,000 at 400 *m*/*z*, and the 12 most intense ions in each cycle were sequentially isolated, fragmented by HCD, and measured in the Orbitrap analyzer. Peptides with a charge of +1 or unassigned charge states were excluded from fragmentation for MS2.

#### 2.7.2 Analysis of mass spectrometry data

Q-Exactive raw data were processed using Proteome Discoverer software (version 2.2 Thermo Scientific) and searched against the *Rattus norvegicus* (Rat) UP000002494_2023_12_11 protein sequence database with trypsin specificity and a maximum of one missed cleavage per peptide. Proteome Discoverer searches were performed with a precursor mass tolerance of 10 ppm and a product ion tolerance of 0.05 Da. Static modifications were set to carbamidomethylation of Cys, and dynamic modifications were set to oxidation of Met and N-terminal acetylation. Protein hits were filtered for high-confidence peptide matches with a maximum protein and peptide FDR of 1% calculated by employing a reverse database strategy. A multi-consensus report was generated using the Proteome Discoverer software. Proteome Discoverer calculates an area for each protein in each condition. To do this, it uses the area under the curve of the three most intense peptides for a protein. Areas were calculated for each of the three replicates and normalized. The data obtained for the area for each protein were processed with the Perseus program (Max Planck Institute of Biochemistry, 1.5.5.3 version, available for free) which allows a deeper statistical analysis. Different scatter plots were prepared according to the compared samples. For each couple of samples, we plotted the log *p*-value (-Log Student’s *t*-test *p*-value A_B) on the *y-axis* against the Student’s *t*-test difference A_B on the *x-axis*. Proteins appear in the volcano plot with a fold change greater than 2 (less than −1 or greater than 1 on the *x-axis* of the graph) and a *p*-value below 0.05 (greater than 1.3 on the *y*-axis of the graph) were considered to be differentially expressed.

### 2.8 Statistical analysis

All statistical analyses were performed using R statistical software (https://www.r-project.org/). Protein abundance was normalized and log2 scaled if required. Quality assessment was performed following Ritchie et al. (22) by applying principal component analysis (PCA) to the samples from each location (**Supplementary Figure S2**).

A linear model was fitted for each protein between conditions for each localization, and an empirical moderated *t*-statistic test for each individual contrast equal to zero was applied by using the limma R package (22). For the cortex-derived samples, the *p*-values were adjusted with the *p-adjust* function. To define differential abundance between conditions, an alpha of 0.05 was selected.

## 3 Results

### 3.1 Proteomics of the cortex

We generated the cortical lesions by injecting AdIL-1β into the cortex, followed by a booster injection of the same adenovector peripherally as described by Silva et al. (16). This model was validated using blood smears, behavioral tests, and anatomopathological studies on the animals utilized for proteomic analysis as previously published (**Supplementary Figures S1**, **S2**). As described in the Materials and Methods section, we studied two groups: one with cortical IL-1β/peripheral IL-1β (experimental group) (IL-1β c/IL-1β iv) (*n* = 5) and the other with cortical Adβ-gal/peripheral Adβ-gal (β-gal c/β-gal iv) (control group) (*n* = 5) administration (**Figure 1**). As expected, animals in the experimental group showed behavioral alterations and an increase in leukocyte blood count (**Supplementary Figure S1**). In addition, we performed some controls for the proteomics, and the IL-1β c/IL-1β iv animals presented a diminished inflammatory infiltration and less glial activation compared with β-gal c/β-gal iv (**Supplementary Figure S2**).

**Figure 2:**
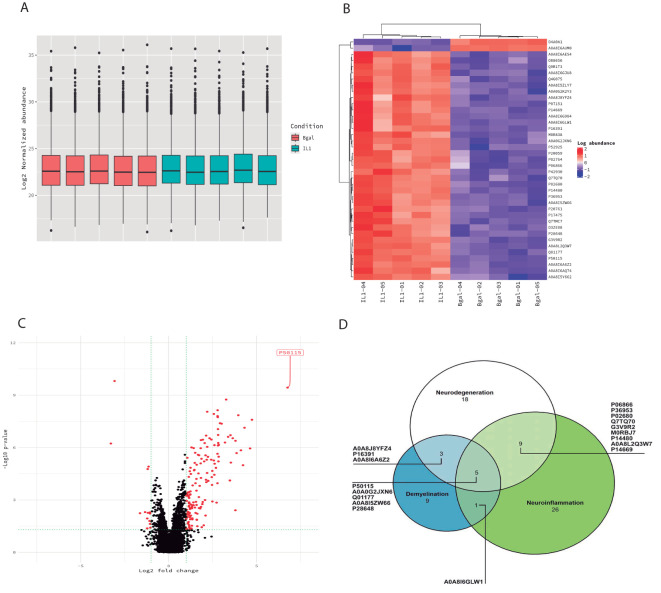
Proteomic profiling of the cortex. **(A)** Box plots of the normalized abundance of the total amount of proteins in both experimental (blue) and control groups (pink). No significant variation in the total protein levels between the cortex of the experimental and control animals. **(B)** Heatmap of the differentially expressed proteins (DEPs) in the cortex of experimental animals compared to control animals. Each column of the heatmap represents one animal and the rows indicate the expression of the DEPs. **(C)** Volcano plot of the DEP proteins in the cortex. **(D)** Venn diagrams showing the number of DEPs in each biological process (neuroinflammation, neurodegeneration, and demyelination).

Proteomic analysis in cortical lesions revealed 5,336 expressed proteins (**Supplementary Data S1**) (**Supplementary Figure 3**). To select the molecules showing the highest differences with respect to the entire dataset, we performed further bioinformatic analysis on those molecules whose expression was highly differential between the two groups. As an initial observation, we found no significant variation in the total protein levels between experimental and control animals in the cortex samples (**Figure 2A**).

Of the 5,336 proteins identified in this study, 45 were found to be discriminatory between the experimental groups (IL-1β c/IL-1β iv vs. β-gal c/β-gal iv) based on highly stringent criteria (*p* < 0.01) (**Figures 2B, C**) (**Table 1**). Additionally, we performed the analysis considering *p* < 0.05, in which case we obtained 62 differentially expressed proteins (**Supplementary Table S1**). We focused the analysis using a stricter criterion with *p* < 0.01. Based on this criterion, out of 45 proteins, 2 were downregulated and 8 have not been described in MS animal models or connected with MS patients. We classified the proteins based on bibliographic searches into three main potential functions in the CNS that are implicated and represent the main findings in pathophysiology studies of MS: neurodegeneration, demyelination, and neuroinflammation. Out of 45 potential proteins, 26 were related to neuroinflammation, 9 were related to demyelination, and 20 were related to neurodegeneration (see **Table 1**, **Figure 2D**). The Venn diagram visualized the overlap of the different processes in which the proteins are involved (**Figure 2D**). Only 10 proteins could not be assigned to any of these important features in the MS cortex of patients, providing a first indication of the validity of the previously described model.

**Table 1:** Differentially expressed proteins in the cortex (*p* < 0.01).

Accession	Description	Log2FC	*p*-value	Adjusted *p*-value	NF	DM	ND
P50115	Protein S100-A8	6.757	<0.001	<0.001	x	x	x
A0A0G2JXN6	Galectin	4.734	<0.001	<0.001	x	x	x
P02764	Alpha-1-acid glycoprotein (orosomucoid)	4.628	<0.001	0.005	x		
Q4G075	Leukocyte elastase inhibitor A	4.311	<0.001	<0.001	x		
P06866	Haptoglobin	4.108	<0.001	0.007	x		x
A0A8I6GJU8	Lymphocyte-specific protein 1	3.960	<0.001	<0.001	x		
A0A8J8YFZ4	Polypyrimidine tract-binding protein 1	3.862	<0.001	0.01		x	x
P16391	RT1 class I histocompatibility antigen, AA alpha chain	3.799	<0.001	0.002		x	x
A0A8I5ZLY7	TAP-binding protein	3.640	<0.001	0.001			
P52925	High-mobility group protein B2	3.514	<0.001	0.001			
A0A8I6G984	Lymphocyte cytosolic protein 1	3.380	<0.001	0.001	x		
A0A8I6A6Z2	Capping actin protein, gelsolin like	3.269	<0.001	<0.001		x	x
A0A8I6GLW1	Allograft inflammatory factor 1	3.201	<0.001	0.002	x	x	
P36953	Afamin	3.008	<0.001	<0.001	x		x
P02680	Fibrinogen gamma chain	2.918	<0.001	<0.001	x		x
P20059	Hemopexin	2.850	<0.001	0.003	x		
O88656	Actin-related protein 2/3 complex subunit 1B	2.817	<0.001	0.003	x		
Q7TQ70	Fibrinogen alpha chain	2.811	<0.001	0.001	x		x
P42930	Heat shock protein beta-1	2.804	<0.001	<0.001			x
A0A8I5Y662	Ig-like domain-containing protein	2.802	<0.001	0.003			
Q01177	Plasminogen	2.791	<0.001	<0.001	x	x	x
G3V9R2	Complement factor H	2.782	<0.001	<0.001	x		x
Q9R1T3	Cathepsin Z	2.778	<0.001	<0.001	x		
M0RBJ7	Complement C3	2.774	<0.001	0.01	x		x
A0A0G2K2Y3	Aminopeptidase	2.602	<0.001	0.003	x		
P14480	Fibrinogen beta chain	2.566	<0.001	<0.001	x		x
D3ZE08	Ig-like domain-containing protein	2.428	<0.001	0.002	x		
M0R838	Ig-like domain-containing protein	2.324	<0.001	0.001			
A0A8L2Q3W7	Pregnancy-zone protein	2.192	<0.001	<0.001	x		x
P07151	Beta-2-microglobulin	2.192	<0.001	0.004			
A0A8I5ZW66	Vitamin D-binding protein	2.129	<0.001	0.001	x	x	x
A0A8I6AQ74	Ig-like domain-containing protein	2.048	<0.001	0.003			
P17475	Alpha-1-antiproteinase	1.943	<0.001	0.002			
Q7TMC7	Signal recognition particle receptor subunit beta	1.838	<0.001	0.002			
P14669	Annexin A3	1.820	<0.001	0.005	x		x
P20761	Ig gamma-2B chain C region	1.507	<0.001	0.009	x		
A0A8I6AES4	Cathepsin S	1.420	<0.001	0.005	x		
P28648	CD63 antigen	1.299	<0.001	0.004	x	x	x
D4A0A1	SOGA family member 3	−3.077	<0.001	<0.001			

The last three columns indicate the function of the proteins in different processes: NF, neuroinflammation; DM, demyelination; ND, neurodegeneration.

Bibliographical analysis revealed that the majority of the differential proteins were related to inflammation, migration, integrin signaling, and protease activity, although we did not further analyze these proteins related to the inflammatory process because their presence is expected since we induced inflammation as part of the model. Nevertheless, again, their identification validates inflammation as the biological process underlying the model.

There are several interesting differential proteins based on both their abundance and their possible function related to the MS pathophysiology. Among them, we found upregulated proteins in experimental animals, such as S100A8, orosomucoid, galectin-3, afamin, hemopexin, pregnancy zone protein, gelsolin, and vitamin D-binding protein. Among the main differential proteins, there are three proteins that are involved in both neurodegeneration and demyelination processes but not in neuroinflammation: gelsolin (GSN), polypyrimidine tract-binding protein 1 (PTB), and RT1 class histocompatibility antigen AA alpha chain (RT1). Therefore, these proteins may be relevant to the pathophysiology independent of the inflammatory process.

### 3.2 Proteomics of the CSF

As an initial observation, we found no significant variation in the total protein levels between experimental and control animals in the CSF samples (**Figure 2A**). Proteomic analysis of the CSF demonstrated the presence of 6,427 relevant proteins.

**Figure 3:**
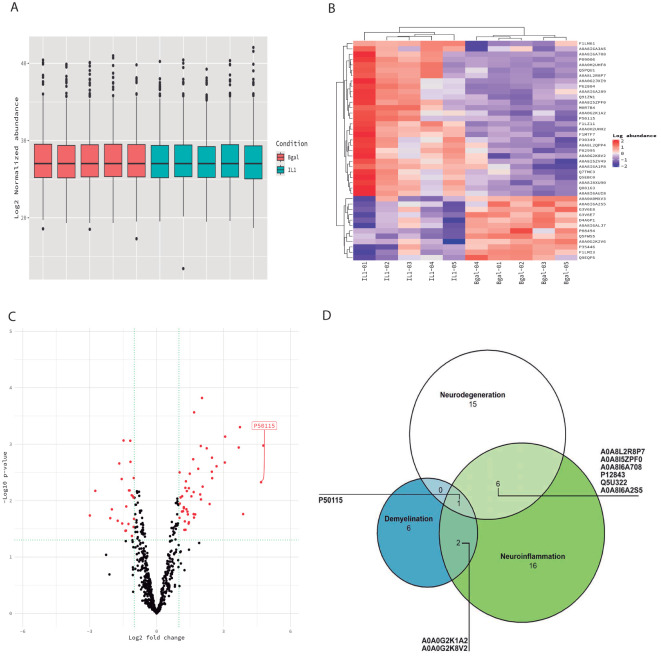
Proteomic profiling of the CSF. **(A)** Box plots of the normalized abundance of the total amount of proteins in both experimental (blue) and control groups (pink). No significant variation in the total protein levels between experimental and control animals in the CSF. **(B)** Heatmap of the differentially expressed proteins (DEPs) in the CSF in experimental animals relative to control animals. Each column of the heatmap represents one animal and the rows indicate the expression of DEPs. **(C)** Volcano plot of the DEP proteins in the CSF. **(D)** Venn diagrams showing the number of DEPs in each biological process (neuroinflammation, neurodegeneration, and demyelination).

Of the 6,427 identified proteins, 48 proteins were differentially expressed between the two experimental groups at *p* < 0.01 (IL-1β c/IL-1β iv vs. β-gal c/β-gal iv) (**Tables 2A**, **B**, **Figure 3**). Out of 48 differential proteins, 16 were downregulated (**Table 2**) (**Figures 3B, C**). We also classified the proteins according to bibliographic research into three different main potential functions: neurodegeneration, demyelination, and neuroinflammation, where 17 proteins were related to neuroinflammation, 7 were related to demyelination, and 15 were related to neurodegeneration (see **Table 2**, **Figure 3D**). The Venn diagram visualized the overlap of the different processes in which the proteins are involved (**Figure 3D**). Out of the 48 proteins, 16 proteins were downregulated and related to the control animals, and 8 proteins were not mentioned in either the MS animal models or those related to MS patients.

**Table 2A:** Differentially expressed proteins upregulated in the CSF (*p* < 0.01).

Accession	Description	Log2 FC	*p*-value	NF	DM	ND
F1LN61	Immunoglobulin heavy constant epsilon	1.030	0.003	x		
A0A8I5ZPF0	Haptoglobin	1.486	0.003	x		x
A0A8I6A708	Ceruloplasmin	1.355	0.007	x		x
P09006	Serine protease inhibitor A3N	1.314	0.005			
A0A8L2R8P7	Kininogen 1	3.072	0.001	x		x
Q5EBC0	Inter-alpha-trypsin inhibitor heavy chain 4	1.147	0.008	x		
Q5PQU1	Kininogen 1	2.475	0.003			
A0A0H2UHF8	Orosomucoid 1	2.686	0.002	x		
G3V6E8	Myocilin	-1.673	0.002			x
Q7TMC3	Hermansky-Pudlak syndrome 5 protein homolog	1.561	0.008			
A0A0H2UHH2	Amyloid P component, serum	1.679	<0.001			
A0A0G2JXI9	Histone H2B	2.482	0.002			
P50115	Protein S100-A8	4.681	0.005	x	x	x
A0A8I6A289	Histone H3	1.661	0.007			
F1M7F7	Complement component C6	1.981	0.001		x	
P82995	Heat shock protein HSP 90-alpha	1.074	0.009			
P30349	Leukotriene A-4 hydrolase	1.446	0.006	x		
A0A8I6A3A5	Ig-like domain-containing protein	1.988	0.01		x	
A0A8I6AUI8	Myosin light chain 6	1.287	0.006			
A0A8I6A1P8	Rac family small GTPase 2	2.491	0.004			x
A0A8J8XU90	Myosin, heavy chain 9	2.246	0.001			
P68255	14-3-3 protein theta	0.927	0.009			x
A0A0G2K8V2	Vinculin	1.955	0.008	x	x	
P62804	Histone H4	3.682	0.001			
A0A8I5ZV49	Filamin A	3.058	0.002			
Q91ZN1	Coronin-1A	2.063	0.006	x		
M0R7B4	H1.3 linker histone, cluster member	3.736	<0.001			
Q08163	Adenylyl cyclase-associated protein 1	1.640	0.002			
A0A8L2QPP4	F-actin-capping protein subunit alpha	1.866	0.003			x
A0A0G2K1A2	Myeloperoxidase	4.784	0.001	x	x	

The last three columns indicate the function of the proteins in different processes NF, Neuroinflammation; DM, Demyelination; ND, Neurodegeneration.

**Table 2B:** Differentially expressed proteins downregulated in the CSF (*p* < 0.01).

Accession	Description	Log2 FC	*p*-value	NF	ND	DM
P12843+B53A38:A53	Insulin-like growth factor-binding protein 2	−0.871	0.007	x	x	
Q5U322	Carboxypeptidase E	−0.991	0.009	x	x	
A0A8I6A2S5	Heparan sulfate proteoglycan 2	−1.115	0.008	x	x	
F1LS40	Collagen type I alpha 2 chain	−0.779	0.008			
D4A6P1	Seizure related 6 homolog like 2	−1.033	0.009	x		
G3V6E7	Fibromodulin	−1.482	0.001		x	
A0A0G2K2V6	Keratin 10	−2.747	0.007			x
A0A8I6ALJ7	SPARC	−1.564	0.004	x		
Q9EQP5	Prolargin	−1.058	0.008		x	
Q5ZQU0	Sushi, nidogen and EGF-like domain-containing protein 1	−0.884	0.007			
Q5FWS5	Lysyl oxidase homolog	−1.180	0.001			
A0A0A0MXV3	Cell growth regulator with EF hand domain 1	−1.208	0.007			
G3V7Y2	Osteomodulin	−0.788	0.007			
F1LMI3	Cadherin 3	−1.024	0.004			
P35446	Spondin-1	−1.239	0.007		x	
P08494	Matrix Gla protein	−1.171	0.002		x	

The last three columns indicate the function of the proteins in different processes: NF, neuroinflammation; DM, demyelination; ND, neurodegeneration.

Differentially expressed proteins were related to acute class I proteins, such as haptoglobin, ceruloplasmin, Orm1 (alpha 1 acid glycoprotein), and components of the complement system. These proteins were upregulated during the ongoing inflammatory processes (23–25).

Interestingly, we found 16 downregulated proteins in the CSF. Among these proteins, we found some of them to be related to the extracellular matrix (lysyl oxidase, spondin-1, fibromodulin, prolargin, collagen type 1 alpha 2 chain, and SPARC). Insulin-like growth factor binding protein 2 was also downregulated and described to be involved in neuroprotective and myelinogenetic effects (26). In addition, in the CSF, S100A8 and Orm1 were differentially expressed, which are analyzed in the Discussion section.

### 3.3 Analysis of the proteomics of cortex and CSF

Linear regression contrasting IL-1β c/IL-1β iv vs. β-gal c/β-gal iv was adjusted to each protein across all samples. Differentially expressed proteins were identified, and those with differential abundance and an adjusted *p* < 0.01 in both regions were then selected.

**Figure 4:**
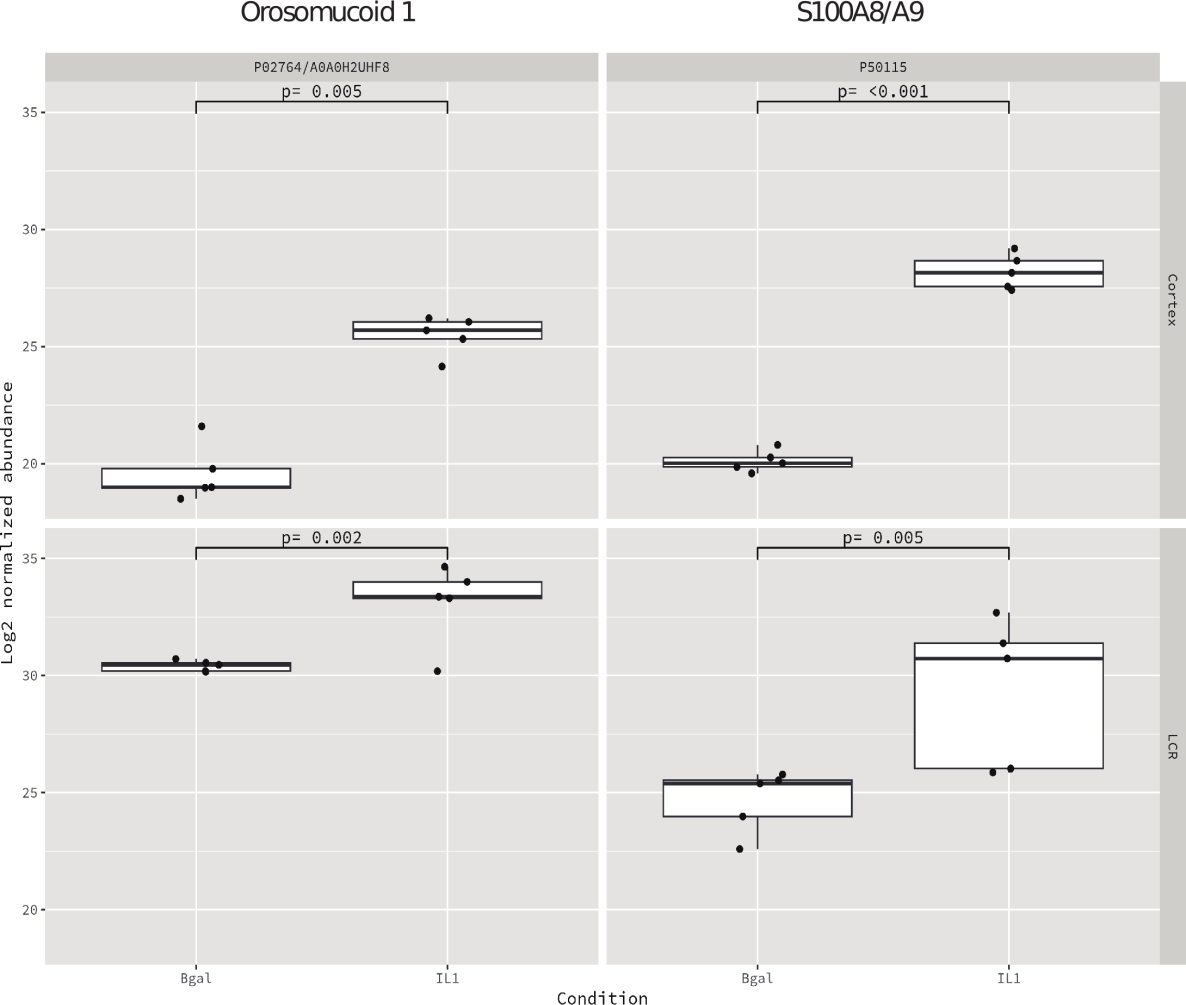
Shared proteins between the cortex and CSF based on the median and IQR with the filter of *p* < 0.01.

PCA was conducted on samples from both regions, revealing that the two experimental conditions clustered appropriately, albeit with a slightly smaller distance than those derived from the CSF (**Figure 4**).

We found two proteins, S100A8/A9 and Orm1, that were highly expressed in both compartments, cortex, and CSF (**Figure 3**) (**Table 3**).

**Table 3:** Differentially expressed proteins in both cortex and CSF (*p* < 0.01).

Accession	Sample	Bgal - Median	Bgal - Q1	Bgal - Q3	IL1 - Median	IL1 - Q1	IL1 - Q3
P02764/A0A0H2UHF8	Cortex	18.996	18.971	19.780	25.691	25.324	26.048
	LCR	30.463	30.195	30.546	33.366	33.306	34.000
P50115	Cortex	20.021	19.856	20.265	28.146	27.559	28.658
	LCR	25.397	23.991	25.530	30.729	26.031	31.387

## 4 Discussion

Our group has developed one of the few animal models that reflects the characteristics of cortical compromise of MS. We have previously described the anatomopathological, behavioral, and radiological characteristics of our model (16). Furthermore, we have demonstrated the cortical effects of repeated peripheral inflammation over long periods of time (27) and the effect of non-pharmacological interventions on these findings (28). In the present work, we propose to study in depth the proteome of the cortical lesion and CSF in the proposed model aiming to identify a molecular expression pattern that could reveal new therapeutic targets and biomarkers involved in cortical compromise. To the best of our knowledge, only a few publications have analyzed the cortical proteome of two other animal models: EAE and cuprizone (18–20).

The proteomic analyses revealed distinct regional differences in this cortical model of MS mediated by the innate immune system that we developed. Differences in the proteome were found between frontal cortex tissue and CSF when comparing experimental animals with control animals. Notably, the induction of the cortical lesions did not lead to an increase in the total amount of proteins in either of the studied areas. Interestingly, despite the presence of different proteins in the two analyzed regions, we identified two proteins that were highly expressed in both areas.

Specifically, our study uncovered that proteins associated with inflammation were upregulated in animals injected with cortical and peripheral AdIL-1β. However, our focus for further analysis will be on proteins that are not strictly linked to the inflammatory process. In this study, we identified several proteins that have already been described either in MS patients or animal models (**Tables 4A**, **B**). However, we also discovered novel molecules that have not been previously reported (**Tables 5A**, **B**). These novel molecules may play a role in mechanisms specifically related to the pathogenesis of cortical lesions. The majority of the proteins identified in both cortex and CSF are involved in inflammatory and neurodegenerative processes, with fewer proteins playing a role in demyelination. These findings suggest that our model more closely mimics the cortical EAE model, which is widely accepted in the scientific community as the model that most closely resembles the pathophysiology of MS, as opposed to classic demyelination models such as cuprizone or lysolecithin.

**Table 4A:** Protein expressed in the cortex linked to other animal models and MS patients.

Accession	Description	Log2FC	*p*-value	Evidence in MS animal models	Evidence in MS patients
P50115	Protein S100-A8	6.757	<0.001	Increased microglial activation and apoptosis of oligodendrocytes *in vitro* (45)	Increased in serum of RRMS (61)
A0A0G2JXN6	Galectin 3	4.734	<0.001	-Cuprizone lysolecithin-induced focal spinal cord (62) -TMEV model (63) -EAE (62, 64)	Increased in SPMS patient sera (65)
P02764	Alpha-1-acid glycoprotein (orosomucoid)	4.628	<0.001	EAE and cuprizone (20)	CSF and serum (53, 55, 56)
Q4G075	Leukocyte elastase inhibitor A	4.311	<0.001	No	No
P06866	Haptoglobin	4.108	<0.001	No	-Serum RRMS (66) -CSF PPMS, SPMS, RRMS (67) -CSF and serum (68, 69), Serum (70). Childhood—MS onset (44) -Meningeal inflammation (71)
A0A8J8YFZ4	Polypyrimidine tract-binding protein 1	3.862	<0.001	TMEV (33)	Tissue SNC (32)
P16391	RT1 class I histocompatibility antigen, AA alpha chain	3.799	<0.001	Cortical EAE (34)	No
A0A8I6GLW1	Allograft inflammatory factor 1	3.201	<0.001	EAE (72)	No
P36953	Afamin	3.008	<0.001	EAE (18)	No
P02680	Fibrinogen gamma chain	2.918	<0.001	EAE (73)	No
P20059	Hemopexin	2.850	<0.001	EAE (19, 36)	Pediatric MS (60)
Q7TQ70	Fibrinogen alpha chain	2.811	<0.001	No	SPMS (74)
P42930	Heat shock protein beta-1 (HSP27)	2.804	<0.001	No	-Serum RRMS (75–77) -CSF (78)
Q01177	Plasminogen	2.791	<0.001	EAE (79–85)	-Genes and MS brains (86–88) -Plasma RRMS and PMS (89, 90) -Blood RRMS (91)
G3V9R2	Complement factor H	2.782	<0.001	EAE (92)	-Serum and CSF (93, 94)
Q9R1T3	Cathepsin Z	2.778	<0.001	EAE (95)	No
M0RBJ7	Complement C3	2.774	<0.001	-EAE (96–100) -EAE and TMEV (101)	-CSF and serum (102, 103) -Postmortem MS brains (97, 104–106) -Plasma RRMS and SPMS (107) -CSF (108–110) -Serum (111, 112)
A0A0G2K2Y3	Aminopeptidase (ERAP 1)	2.602	<0.001	EAE (113)	Genes RRMS (114)
P14480	Fibrinogen beta chain	2.566	<0.001	No	-Blood SPMS (74) -Serum RRMS (94)
P07151	Beta-2-microglobulin	2.192	<0.001	TMEV (115) EAE (116, 117)	-CSF RRMS (118–121) -CSF and serum RRMS and PMS (122–124)
A0A8I5ZW66	Vitamin D-binding protein	2.129	<0.001	EAE (125)	-Serum pediatric MS (60) -CSF PMS (125, 126) -Serum RRMS (127)
P17475	Alpha-1-antiproteinase/antitrypsin	1.943	<0.001	EAE (128)	-CSF RRMS (129) -Serum RRMS (130, 131)

RRMS, Relapsing remitting multiple sclerosis; PPMS, Primary progressive multiple sclerosis; SPMS, secondary progressive multiple sclerosis.

**Table 4B:** Protein expressed in the CSF linked to other animal models and MS patients.

Accession	Description	Log2FC	*p*-value	Evidence in MS animal models	Evidence in MS patients
A0A8L2R8P7	Kininogen 1	3.072	0.001	EAE CSF (18)	Pediatric MS (60, 132)
Q5PQU1	Kininogen 1	2.475	0.003	EAE CSF (18)	Pediatric MS (60)
A0A0H2UHF8	Orosomucoid 1	2.686	0.002	EAE and Cuprizone (20)	Plasma and CSF of MS patients (53)
A0A0H2UHH2	Amyloid P component, serum	1.679	<0.001	EAE (133, 134)	Pediatric MS (60)
A0A0G2JXI9	Histone H2B	2.482	0.002	EAE (135)	Plasma of MS patients (136, 137)
P50115	Protein S100-A8	4.681	0.005	Increased microglia activation and apoptosis of oligodendrocytes *in vitro* (54)	CSF RRMS (60, 138)
A0A8I6A289	Histone H3	1.661	0.007	EAE, cuprizone (135, 139)	MS (136, 137)
F1M7F7	Complement component C6	1.981	0.001	EAE, cuprizone (140)	Serum and CSF (99, 140, 141)
P62804	Histone H4	3.682	0.001	EAE, cuprizone (135, 139)	MS (136, 137)
Q91ZN1	Coronin-1A	2.063	0.006	EAE (142)	MS (143)
A0A0G2K1A2	Myeloperoxidase	4.784	0.001	EAE (144)	MS (145)
A0A8L2R8P7	Kininogen 1	3.072	0.001	In EAE CSF (22)	Pediatric MS (60, 132)
A0A0H2UHF8	Orosomucoid 1	2.686	0.002	EAE, cuprizone (20)	CSF and serum of MS patients (53)
A0A0G2K2V6	Keratin 10	−2.747	0.007	Lysophosphatidyl choline (*in vitro*) (LPC) (146)	no
A0A8I6ALJ7	SPARC	−1.564	0.004	No	MS patients (147)

**Table 5A:** Proteins differentially expressed in the cortex that have not been previously linked to other animal models or MS patients.

Accession	Description	Log2FC	*p*-value	Evidence in MS animal models	Evidence in MS patients
A0A8I6GJU8	Lymphocyte-specific protein 1	3.960	<0.001	No	No
A0A8I5ZLY7	TAP binding protein	3.640	<0.001	No	No
P52925	High-mobility group protein B2	3.514	<0.001	No	No
A0A8I6G984	Lymphocyte cytosolic protein 1	3.380	<0.001	No	No
A0A8I6A6Z2	Capping actin protein, gelsolin like	3.269	<0.001	No	No
O88656	Actin-related protein 2/3 complex subunit 1B	2.817	<0.001	No	No
A0A8I5Y662	Ig-like domain-containing protein	2.802	<0.001	No	No
D3ZE08	Ig-like domain-containing protein	2.428	<0.001	No	No
M0R838	Ig-like domain-containing protein	2.324	<0.001	No	No
A0A8L2Q3W7	Pregnancy-zone protein	2.192	<0.001	No	No
A0A8I6AQ74	Ig-like domain-containing protein	2.048	<0.001	No	No
Q7TMC7	Signal recognition particle receptor subunit beta	1.838	<0.001	No	No
P14669	Annexin A3	1.820	<0.001	No	No
P20761	Ig gamma-2B chain C region	1.507	<0.001	No	No
D4A0A1	SOGA family member 3	−3.077	<0.001	No	No
A0A8I6AUM0	DDHD domain containing 2	−3.286	<0.001	No	No

**Table 5B:** Proteins differentially expressed in the CSF that have not been previously linked to other animal models or MS patients.

Accession	Description	Log2FC	*p*-value	Evidence in MS animal models	Evidence in MS patients
A0A8I6A3A5	Ig-like domain-containing protein	1.988	0.01	No	No
A0A8I6A1P8	Rac family small GTPase 2	2.491	0.004	No	No
A0A8J8XU90	Myosin, heavy chain 9	2.246	0.001	No	No
A0A0G2K8V2	Vinculin	1.955	0.008	No	No
A0A8I5ZV49	Filamin A	3.058	0.002	No	No
M0R7B4	H1.3 linker histone, cluster member	3.736	<0.001	No	No
Q08163	Adenylyl cyclase-associated protein 1	1.640	0.002	No	No
A0A8L2QPP4	F-actin-capping protein subunit alpha	1.866	0.003	No	No

In the cortex of experimental animals, we identified 45 proteins that met highly stringent criteria for discrimination. The majority of these proteins are associated with the pathophysiology of multiple sclerosis (MS), particularly in processes related to neurodegeneration, demyelination, and neuroinflammation (**Tables 1**, **2**). In the cortex analyses, three proteins were found to be implicated in both neurodegeneration and demyelination processes, but not in neuroinflammation, namely, gelsolin (GSN), polypyrimidine tract-binding protein 1 (PTB), and RT1 class histocompatibility antigen AA alpha chain (RT1). Therefore, these proteins may be relevant to the pathophysiology independent of the inflammatory process. Gelsolin is the fourth most abundant protein in the body and its depletion in the blood has been found in MS patients. The secreted form of gelsolin (pGSN) decreased in the blood of EAE mice, and pGSN concentration increased in the EAE brain (29, 30). In addition, gelsolin works downstream of the LINGO-1 signaling pathway, which enhances actin dynamics and is essential for oligodendrocyte precursor cells’ morphogenesis and differentiation (31). PTB is important for neuronal differentiation, suggesting that this molecule may contribute to the pathogenesis of demyelination and neurodegeneration in MS lesions and *in vitro* cultured primary human brain-derived oligodendrocytes (32). PTB was found to be mislocalized in the Theiler murine encephalomyelitis virus (TMEV)-infected cells (33). Finally, cortical demyelination was exclusively observed in EAE-susceptible non-major histocompatibility complex (MHC)-congenic LEW rat strains and EAE rat strains with a permissive LEW[U2] [C3]. Specifically, cortical demyelination was identified solely in EAE rats of the LEW.1AR1 (RT1) and LEW.1W (RT1) strains (34, 35). We also found interesting molecules that are involved in the pathogenesis of the cortical lesions. Among them, Afamin, a member of the albumin family, was found to be upregulated in rat EAE, along with vitamin D-binding protein, which was found to be upregulated in rat EAE and the CSF of patients with progressive MS (18, 36). Interestingly, in the cortex, we found the pregnancy zone protein (PZP) with a criterion of *p* < 0.05 (see **Supplementary Table S1**). PZP was found in the serum of subjects who later developed Alzheimer’s disease (AD) in comparison to controls who remained dementia-free. PZP immunoreactivity was localized to microglial cells in the cortex of AD patients that interacted with senile plaques and was occasionally observed in neurons (37). The latter two proteins, which have been found to be related to dementia, may be involved in the cognitive impairment observed in MS patients with cortical lesions. Specifically, the proteome of the frontal cortex has been characterized in a few publications that included two MS animal models (cuprizone and EAE). The authors found that Legumain and C1q complement proteins were upregulated in the cuprizone model and hemopexin in the EAE model. Cuprizone is a model of re- and demyelination, and EAE is a T cell-mediated model. However, our model is based on innate immunity, and the animals only exhibited cortical lesions, making it a very clean model to study these lesions. In our study, we have found upregulation in some of the proteins that were described in the previous models, such as hemopexin, which we found upregulated in the cortical tissue. Hemopexin is an acute-phase protein synthesized by hepatocytes in response to the pro-inflammatory cytokines IL-6, IL-1β, and TNF-α, which was described in pediatric MS patients and several MS animal models (38). The expression of this protein could be upregulated in our model because we generated the cortical lesions based on IL-1β injection into the cortex, which, in turn, upregulated IL-6 and TNF-α (16).

In the case of CSF, there were no proteins found to be involved in both demyelination and neurodegeneration without neuroinflammation. Differential upregulated proteins, such as haptoglobin, ceruloplasmin, Orm1 (alpha 1 acid glycoprotein), and components of the complement system were related to the acute class I protein, which was upregulated during the ongoing inflammatory processes (39). The proteomic study of the CSF from EAE animals demonstrated that some of the previous proteins were also upregulated in the rat EAE model: among them, haptoglobin, orosomucoid, ceruloplasmin, and T-kininogen (18). Recently, Barriola et al. (2023) demonstrated that Orm1 was upregulated in two different models (cuprizone and mouse EAE) and two different regions (dorsal cortex and the entire spinal cord) (20). As mentioned above, Orm1 was one of the proteins mainly upregulated in both the CSF and frontal cortex in our model. Interestingly, some of the downregulated proteins in the CSF were related to the extracellular matrix: matrix (lysyl oxidase, spondin-1, fibromodulin, prolargin, collagen type 1 alpha 2 chain, and SPARC). The protein insulin-like growth factor binding protein 2 has been described to be involved in neuroprotective and myelinogenetic effects (40), and has been found to be upregulated in the microglia/macrophages in active MS lesions (41). However, it was not upregulated in the CSF of MS patients (42); carboxypeptidase E has been described to be upregulated in pediatric MS patients (43, 44).

We also performed a further analysis in order to search which proteins were expressed in both compartments, cortex and CSF. Two proteins, S100A8 and Orm1, were upregulated in both compartments with the strict criterion of *p* < 0.01.

S100A8 is a Ca^2+^ binding protein belonging to the S100 family. This protein is constitutively expressed in neutrophils and monocytes. During the inflammatory process, S100A8 is actively released and exerts a crucial role in modulating inflammation by stimulating leukocyte recruitment and inducing cytokine expression (45). Pro-inflammatory cytokines, such as TNF-α and IL-1β, induce its transcription (46). Therefore, this protein could be considered as a candidate biomarker and as an indicator of response to inflammation-associated disease. The expression of S100A8/A9 in this model may be related to the presence of neutrophils induced by IL-1β, which characterizes this model (16). As previously described, the model is based on neutrophil recruitment as the first symptom; this recruitment of neutrophils decreases over time. The neutrophil wave is followed by an increase in microglia/monocyte recruitment (16). Recently, the role of neutrophils in the pathogenesis of MS has begun to be studied, especially considering that these immune cells have been underestimated in the context of MS (47). The presence of neutrophils has been described as a trigger of the inflammatory process in the EAE and JHM strains of the mouse hepatitis virus demyelinating model and in the cuprizone model (48, 49). It has been proposed that the neutrophils contribute to EAE pathology by disrupting the BBB, as occurred in our model, in which neutrophils contributed to BBB breakdown (16, 50). Neutrophils accumulated in the meninges of EAE mice during the preclinical and peak stages, as described in our AdIL-1β induced model. Even though neutrophils were among the last immune cell types described to be involved in the pathogenesis of MS, nowadays, the role of neutrophils in MS patients is still not fully understood, but research is still ongoing in order to elucidate their function in MS disease. Neutrophils have been found in the CSF in both adult and pediatric MS patients during relapses and in the early disease stages (47, 51, 52). At present, research is focused on elucidating whether neutrophils can be proposed as biomarkers and whether the manipulation of neutrophils can be used in future MS treatments (53).

S100A8 is significantly increased in the serum of RRMS patients (53). Indeed, it has been demonstrated that S100A8/A9 treatment induces activation, proliferation, and migration of the murine microglial cell line BV-2, which changes the microglial state from an anti-inflammatory state (M2) to a pro-inflammatory phenotype (M1) (54). Interestingly, S100A8 has not been described in any animal model of MS.

Orm1, also known as alpha 1 acid glycoprotein, acts as a transport protein in the bloodstream and modulates the activity of the immune system during the acute-phase reaction and is a glycoprotein mainly formed in the liver; it is also highly expressed in the frontal cortex (55). The expression of the Orm1 gene is mainly controlled by glucocorticoids and interleukins (IL-1β, TNF-α, and IL-6). It is interesting to mention that these pro-inflammatory proteins were also found to be upregulated in our model (16). Orm1 was found to be altered in two different regions, the spinal cord and frontal cortex, in two different models, EAE and cuprizone (20). Orm1 has been described in the CSF, serum, and plasma of MS patients (53, 56–60). Recently, a study focused on two different regions, the dorsal cortex, and the entire spinal cord, in two different models (EAE and cuprizone), and the authors identified Orm1 as a single protein that consistently exhibited alterations in both models and regions (23).

Finally, in the present work, we identified molecules that were upregulated or downregulated in both cortical tissue and CSF in other animal models of MS and that were also analyzed as biomarkers of MS development or disease progression in the blood, CSF, and brain tissue of MS patients (**Tables 4**, **5**). Some of them, such as haptoglobin, hsp 27, plasminogen, complement C3, beta 2 microglobulin, and vitamin D-binding protein, are in more advanced phases of research and their relevance as biomarkers has been demonstrated in many studies (**Table 4**). On the other hand, our work provided a large number of new cortical and CSF molecules not found in other animal or patient studies of MS (**Table 5**). This work opens the door to the search for new therapeutic targets and MS biomarkers of cortical involvement.

The advantage of this model is its exclusive focus on frontal cortex lesions. Consequently, this study not only validates the model as a novel representation of MS cortical lesions but also suggests that some of the identified molecules could serve as potential biomarkers for MS, particularly in forms where cortical lesions are the predominant pathophysiological feature. Again, investigating molecules that have not been previously associated with MS pathology could be valuable in the search for novel prognostic markers or biomarkers. The translation of prognostic molecules from animal models to MS patients is crucial, as it enables the discovery of new biomarkers or key molecules that would be challenging to identify in human studies. Further validation studies will be necessary to identify specific target molecules that may be involved in each form of MS.

## Data Availability

The database generated for this study data has been deposited to the PRIDE repository with the dataset identifier PXD050417.7.2.

## Supplementary material

Supplementary Figure 1
**(A)** Blood smear. Blood smears were performed 5 days after the peripheral injection of AdIL-1β and Adβ-gal Representative blood smear counts as a control for the effectiveness of peripheral stimulus (Unpaired t-test) (n = 8/group).**p < 0.001–0.01 L, lymphocytes; N, neutrophils. **(B)** Locomotor activity and anxious behavior. Line crossings [**(B (A)**], time in the center **[(B (B)]**, and rearings [**(B (C)**] were significantly decreased by 7 dpi in animals with peripheral inflammation compared with animals without peripheral inflammation. Furthermore, fecal boli [**(B (D)**] compared with animals without peripheral inflammation (peripheral βgal). Unpaired t test, n = 8/10 group, * p < 0.01–0.05 ***p < 0.0001–0.001, ****p < 0.0001. **(C)** Short-term memory impairment. Peripherally stimulated with AdIL-1β exhibited short-term memory impairment compared with the control Adβgal animals. Unpaired t-test, n = 8/10 group, **p 0.001–0.01, ***p < 0.0001–0.001. **(D)** Anxiety measured with elevated plus maze test. IL1β peripherally stimulated animals showed a decrease in the percentage of entries [**(D (A)**] and time spent in the open arms [**(D (B)**]. Unpaired t test, n = 8/10 group,* p < 0.01–0.05.

Supplementary Figure 2 Inflammatory infiltrate. **(A-C)** The animals IL-1β c/IL-1β iv have an evident inflammatory infiltrate compared with the control animals by Cresyl Violet staining **(A)**. The inflammatory infiltrate in the IL-1β c/IL-1β iv group was mainly composed of neutrophils (*) and macrophages **(B)**. No inflammatory infiltrate could be observed in Adbgal c/Adbgal iv animals **(C)**. Scale bar: A-C: 200mm. Glial activation. **(D-G)**. Microglial activation in the IL-1β c/IL-1β iv animals was evident compared with the control animals by MHCII **(D, E)**, ED1 **(F, G)** immunohistochemistry. **(H, I)**. Additionally, astroglial activation as demonstrated by GFAP was evident in IL-1β c/IL-1β iv animals **(H)** compared with Adbgal c/Adbgal iv animals **(I)**. Scale bar: 50mm.

Supplementary Figure 3
**(A)** Volcano plot of the abundances of normalized cortical proteins. **(B)** Heatmap of the normalized abundances of cortical proteins.
