# Supplementary figures and images for: Proteomic analysis reveals candidate molecules to mediate cortical pathology and identify possible biomarkers in an animal model of multiple sclerosis
Source: Front Immunol. 2025 Feb 13;16:1505459. doi: 10.3389/fimmu.2025.1505459 (PMC11864942; doi:10.3389/fimmu.2025.1505459)

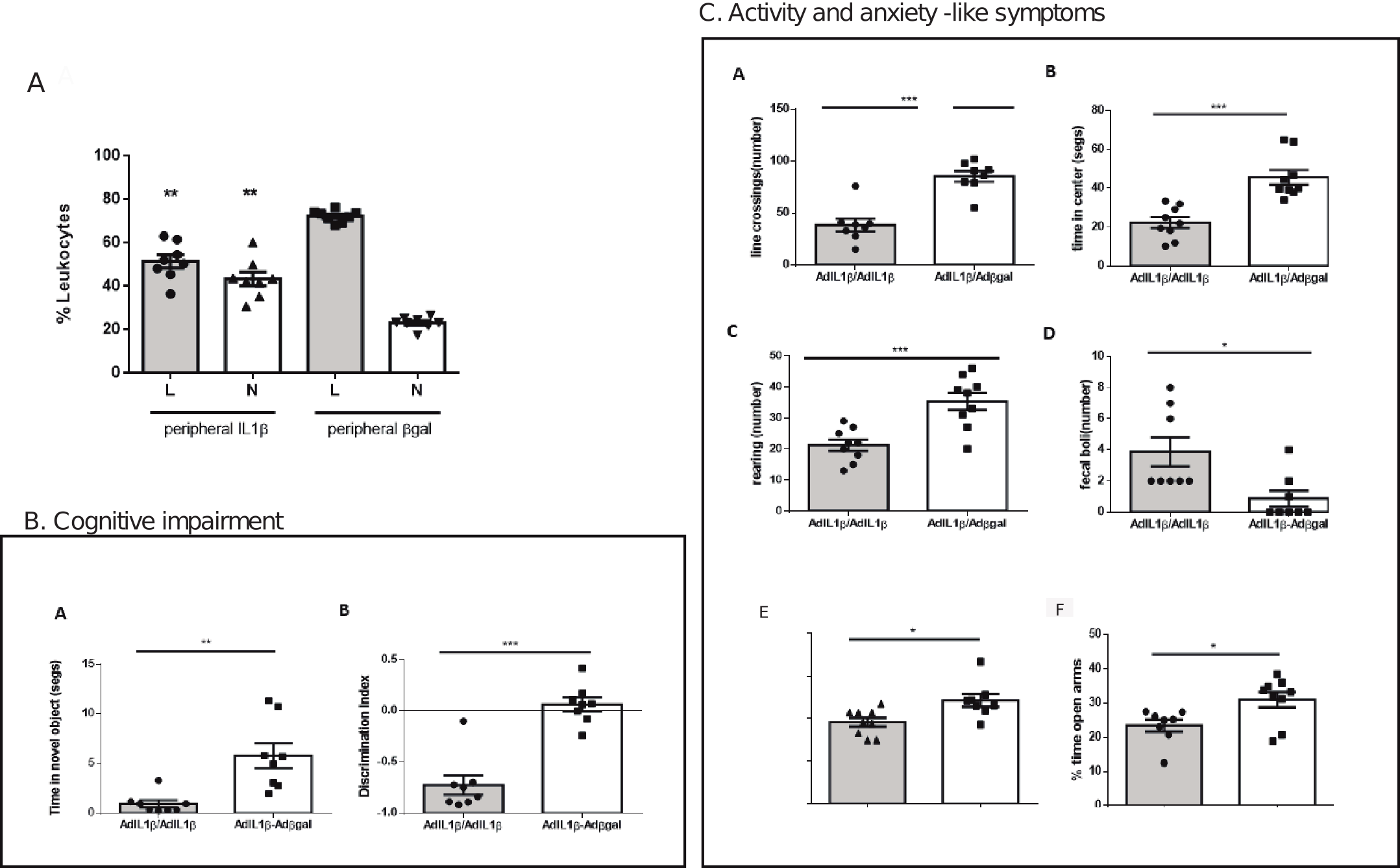

Supplement: Supplementary Figure 1 — (A) Blood smear. Blood smears were performed 5 days after the peripheral injection of AdIL-1β and Adβ-gal Representative blood smear counts as a control for the effectiveness of peripheral stimulus (Unpaired t-test) (n = 8/group).**p < 0.001–0.01 L, lymphocytes; N, neutrophils. (B) Locomotor activity and anxious behavior. Line crossings [(B (A)], time in the center [(B (B)], and rearings [(B (C)] were significantly decreased by 7 dpi in animals with peripheral inflammation compared with animals without peripheral inflammation. Furthermore, fecal boli [(B (D)] compared with animals without peripheral inflammation (peripheral βgal). Unpaired t test, n = 8/10 group, * p < 0.01–0.05 ***p < 0.0001–0.001, ****p < 0.0001. (C) Short-term memory impairment. Peripherally stimulated with AdIL-1β exhibited short-term memory impairment compared with the control Adβgal animals. Unpaired t-test, n = 8/10 group, **p 0.001–0.01, ***p < 0.0001–0.001. (D) Anxiety measured with elevated plus maze test. IL1β peripherally stimulated animals showed a decrease in the percentage of entries [(D (A)] and time spent in the open arms [(D (B)]. Unpaired t test, n = 8/10 group,* p < 0.01–0.05. [file Image1.tiff]

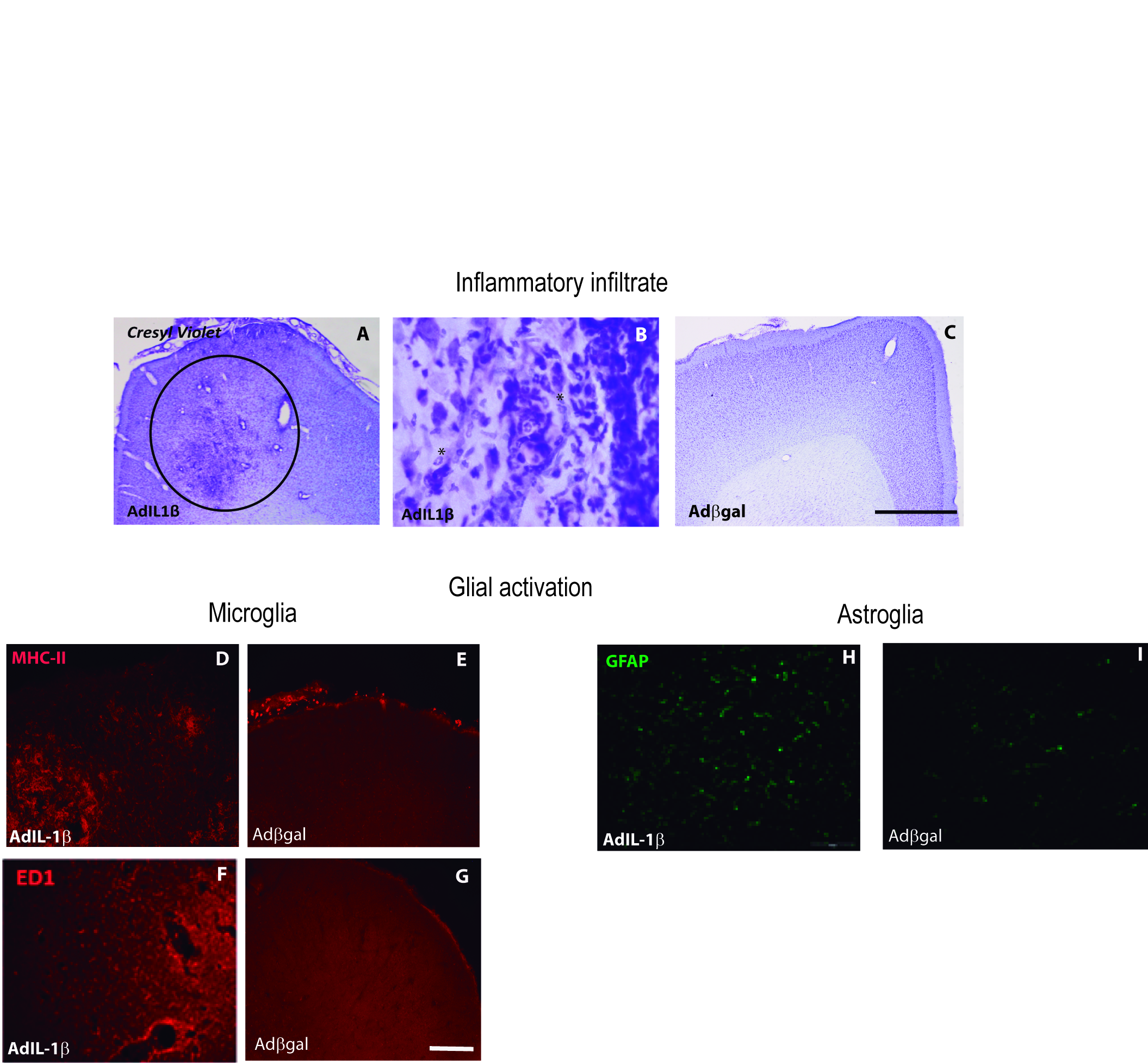

Supplement: Supplementary Figure 2 — Inflammatory infiltrate. (A-C) The animals IL-1β c/IL-1β iv have an evident inflammatory infiltrate compared with the control animals by Cresyl Violet staining (A). The inflammatory infiltrate in the IL-1β c/IL-1β iv group was mainly composed of neutrophils (*) and macrophages (B). No inflammatory infiltrate could be observed in Adbgal c/Adbgal iv animals (C). Scale bar: A-C: 200mm. Glial activation. (D-G). Microglial activation in the IL-1β c/IL-1β iv animals was evident compared with the control animals by MHCII (D, E), ED1 (F, G) immunohistochemistry. (H, I). Additionally, astroglial activation as demonstrated by GFAP was evident in IL-1β c/IL-1β iv animals (H) compared with Adbgal c/Adbgal iv animals (I). Scale bar: 50mm. [file Image2.tif]

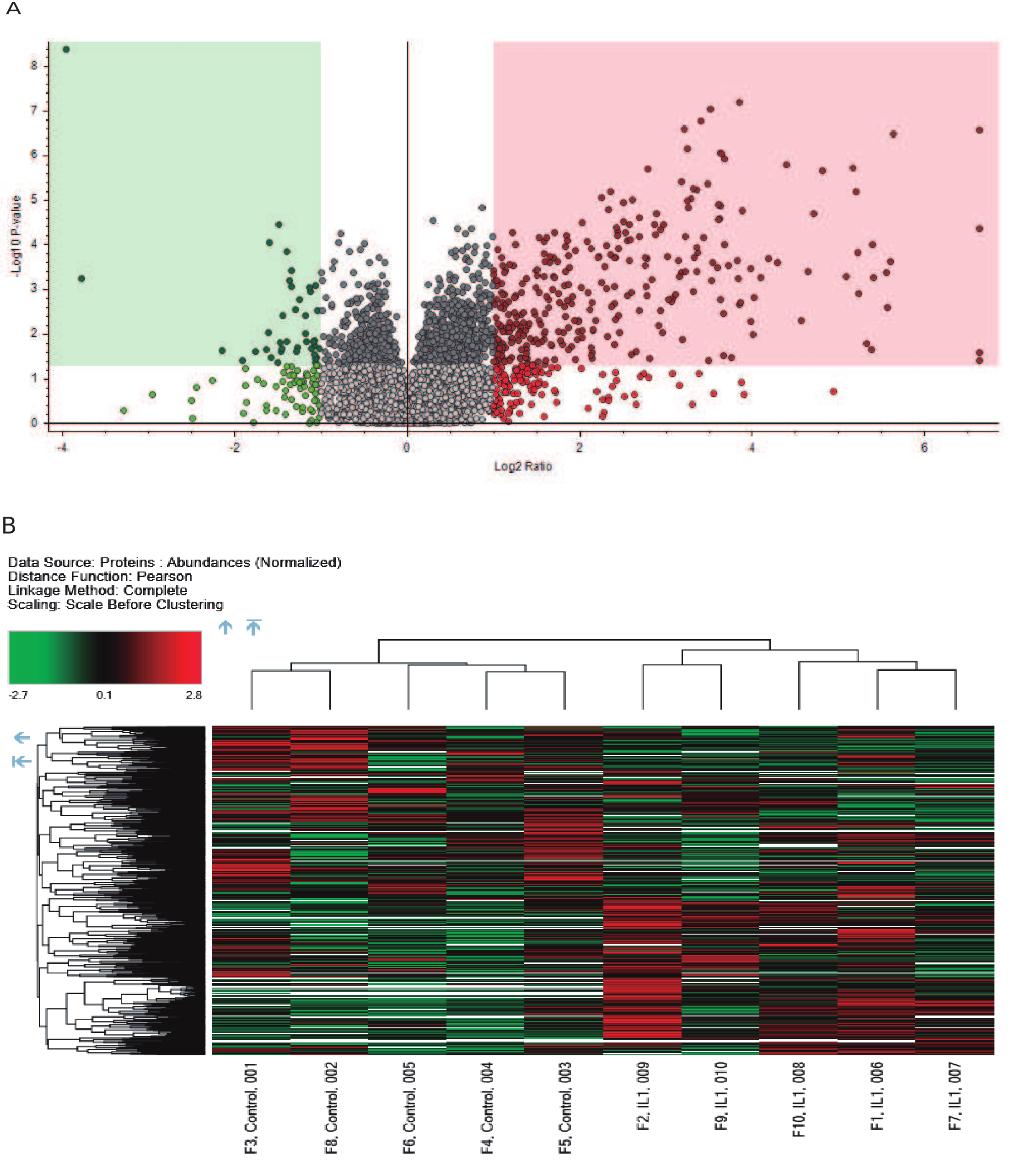

Supplement: Supplementary Figure 3 — (A) Volcano plot of the abundances of normalized cortical proteins. (B) Heatmap of the normalized abundances of cortical proteins. [file Image3.tiff]
