# Supplementary material for: Proteomic analysis reveals candidate molecules to mediate cortical pathology and identify possible biomarkers in an animal model of multiple sclerosis
Source: Front Immunol. 2025 Feb 13;16:1505459. doi: 10.3389/fimmu.2025.1505459 (PMC11864942; doi:10.3389/fimmu.2025.1505459)
Supplement: Supplementary file 4 [file Table1.docx]

**Supplementary Table 1.** Differential expressed proteins in the cortex (p<0.05).

| **Accession** | **Description** | **Log2FC** | **P-value** | **Adjusted p-value** |
| --- | --- | --- | --- | --- |
| D4A0A1 | SOGA family member 3 | -3.077 | <0.001 | <0.001 |
| A0A8I6A6Z2 | Capping actin protein, gelsolin like | 3.269 | <0.001 | <0.001 |
| G3V9R2 | Complement factor H | 2.782 | <0.001 | <0.001 |
| P14480 | Fibrinogen beta chain | 2.566 | <0.001 | <0.001 |
| P50115 | Protein S100-A8 | 6.757 | <0.001 | <0.001 |
| Q01177 | Plasminogen | 2.791 | <0.001 | <0.001 |
| P02680 | Fibrinogen gamma chain | 2.918 | <0.001 | <0.001 |
| A0A8L2Q3W7 | Pregnancy-zone protein | 2.192 | <0.001 | <0.001 |
| A0A0G2JXN6 | Galectin | 4.734 | <0.001 | <0.001 |
| A0A8I6GJU8 | Lymphocyte-specific protein 1 | 3.960 | <0.001 | <0.001 |
| Q4G075 | Leukocyte elastase inhibitor A | 4.311 | <0.001 | <0.001 |
| P52925 | High mobility group protein B2 | 3.514 | <0.001 | 0.001 |
| Q7TQ70 | Fibrinogen alpha chain | 2.811 | <0.001 | 0.001 |
| Q9R1T3 | Cathepsin Z | 2.778 | <0.001 | <0.001 |
| A0A0G2K2Y3 | Aminopeptidase | 2.602 | <0.001 | 0.003 |
| O88656 | Actin-related protein 2/3 complex subunit 1B | 2.817 | <0.001 | 0.003 |
| A0A8I5ZW66 | Vitamin D-binding protein | 2.129 | <0.001 | 0.001 |
| P20059 | Hemopexin | 2.850 | <0.001 | 0.003 |
| P36953 | Afamin | 3.008 | <0.001 | <0.001 |
| P42930 | Heat shock protein beta-1 | 2.804 | <0.001 | <0.001 |
| D3ZE08 | Ig-like domain-containing protein | 2.428 | <0.001 | 0.002 |
| A0A8I5ZLY7 | TAP binding protein | 3.640 | <0.001 | 0.001 |
| A0A8I6APC0 | Immunoglobulin heavy constant epsilon | 2.232 | <0.001 | 0.016 |
| M0R838 | Ig-like domain-containing protein | 2.324 | <0.001 | 0.001 |
| F1LNT8 | Vesicle-associated membrane protein 8 | 1.865 | <0.001 | 0.02 |
| A0A8I6A5C7 | Proteasome (Prosome, macropain) 28 subunit, beta, isoform CRA_a | 2.164 | <0.001 | 0.023 |
| P02764 | Alpha-1-acid glycoprotein | 4.628 | <0.001 | 0.005 |
| A0A0G2JZI2 | Glutamyl-prolyl-tRNA synthetase | 0.923 | <0.001 | 0.012 |
| F1M853 | Ribosome binding protein 1 | 1.671 | <0.001 | 0.012 |
| P28648 | CD63 antigen | 1.299 | <0.001 | 0.004 |
| P01836 | Ig kappa chain C region, A allele | 2.019 | <0.001 | 0.028 |
| A0A8L2QVV5 | Dolichyl-diphosphooligosaccharide--protein glycosyltransferase subunit 2 | 0.926 | <0.001 | 0.028 |
| A0A8I6AQI7 | Serpin family F member 2 | 2.567 | <0.001 | 0.025 |
| A0A8I6AES4 | Cathepsin S | 1.420 | <0.001 | 0.005 |
| P02651 | Apolipoprotein A-IV | 2.096 | <0.001 | 0.013 |
| A0A8I6AQ74 | Ig-like domain-containing protein | 2.048 | <0.001 | 0.003 |
| A0A8I5Y525 | dolichyl-diphosphooligosaccharide--protein glycotransferase | 1.728 | <0.001 | 0.013 |
| A0A0G2KB85 | Similar to BC049975 protein | 2.614 | <0.001 | 0.038 |
| A0A8I5Y662 | Ig-like domain-containing protein | 2.802 | <0.001 | 0.003 |
| Q7TMC7 | Signal recognition particle receptor subunit beta | 1.838 | <0.001 | 0.002 |
| P20761 | Ig gamma-2B chain C region | 1.507 | <0.001 | 0.009 |
| M0RBJ7 | Complement C3 | 2.774 | <0.001 | 0.01 |
| E9PU28 | Inosine-5'-monophosphate dehydrogenase 2 | 0.924 | <0.001 | 0.035 |
| P14669 | Annexin A3 | 1.820 | <0.001 | 0.005 |
| P07151 | Beta-2-microglobulin | 2.192 | <0.001 | 0.004 |
| Q9QX79 | Fetuin-B | 1.818 | <0.001 | 0.037 |
| P17475 | Alpha-1-antiproteinase | 1.943 | <0.001 | 0.002 |
| A0A8J8YFZ4 | Polypyrimidine tract-binding protein 1 | 3.862 | <0.001 | 0.01 |
| A0A8I6A4U0 | Ig-like domain-containing protein | 2.417 | <0.001 | 0.022 |
| P14046 | Alpha-1-inhibitor 3 | 1.482 | <0.001 | 0.034 |
| A0A8I6G984 | Lymphocyte cytosolic protein 1 | 3.380 | <0.001 | 0.001 |
| P37397 | Calponin-3 | 1.062 | <0.001 | 0.018 |
| Q5EBC0 | Inter alpha-trypsin inhibitor, heavy chain 4 | 2.843 | <0.001 | 0.015 |
| P16391 | RT1 class I histocompatibility antigen, AA alpha chain | 3.799 | <0.001 | 0.002 |
| A0A8I6GLW1 | Allograft inflammatory factor 1 | 3.201 | <0.001 | 0.002 |
| P06866 | Haptoglobin | 4.108 | <0.001 | 0.007 |
| A0A8L2QSI9 | Chloride intracellular channel protein | 3.536 | <0.001 | 0.033 |
| A0A8I6AUM0 | DDHD domain containing 2 | -3.286 | <0.001 | 0.003 |
| P05942 | Protein S100-A4 | 2.829 | <0.001 | 0.028 |
| Q6P9V7 | Proteasome (Prosome, macropain) activator subunit 1 | 1.593 | <0.001 | 0.044 |
| A0A8I5ZK39 | Kininogen 2 | 4.229 | <0.001 | 0.047 |

**Supplementary Table 2**. Differential expressed proteins in the CSF (p<0.05).

| **Accession** | **Description** | **Log2 FC** | **P-value** |
| --- | --- | --- | --- |
| P00762 | Serine protease 1 | -0.743 | 0.016 |
| A0A0G2KB42 | Clusterin | -0.524 | 0.044 |
| P22057 | Prostaglandin-H2 D-isomerase | -0.870 | 0.029 |
| Q64610 | Ectonucleotide pyrophosphatase/phosphodiesterase family member 2 | -0.749 | 0.015 |
| M0RBJ7 | Complement C3 | 0.921 | 0.017 |
| P20059 | Hemopexin | 1.026 | 0.02 |
| Q68FP1 | Gelsolin | -0.524 | 0.039 |
| F1LN61 | Immunoglobulin heavy constant epsilon | 1.030 | 0.003 |
| A0A8I5ZPF0 | Haptoglobin | 1.486 | 0.003 |
| P01836 | Ig kappa chain C region, A allele | 0.666 | 0.031 |
| A0A8I6A708 | Ceruloplasmin | 1.355 | 0.007 |
| P09006 | Serine protease inhibitor A3N | 1.314 | 0.005 |
| P06399 | Fibrinogen alpha chain | 1.447 | 0.024 |
| G3V6X7 | ProSAAS | -0.702 | 0.036 |
| P68035 | Actin, alpha cardiac muscle 1 | 0.906 | 0.027 |
| A0A8L2R8P7 | Kininogen 1 | 3.072 | 0.001 |
| P12843 | Insulin-like growth factor-binding protein 2 | -0.871 | 0.007 |
| P02680 | Fibrinogen gamma chain | 1.297 | 0.033 |
| Q5EBC0 | Inter-alpha-trypsin inhibitor heavy chain 4 | 1.147 | 0.008 |
| A0A0G2K3K2 | Actin, beta | 0.932 | 0.027 |
| A0A1W2Q6Q0 | Osteoglycin | -0.649 | 0.033 |
| Q5U322 | Carboxypeptidase E | -0.991 | 0.009 |
| P14480 | Fibrinogen beta chain | 1.676 | 0.02 |
| G3V615 | C3/C5 convertase | 0.836 | 0.028 |
| A0A8I6A2S5 | Heparan sulfate proteoglycan 2 | -1.115 | 0.008 |
| A0A8I6A5G9 | GM2 ganglioside activator | -0.687 | 0.015 |
| F7F389 | Complement C9 | 1.134 | 0.016 |
| P02625 | Parvalbumin alpha | -1.371 | 0.034 |
| Q5PQU1 | Kininogen 1 | 2.475 | 0.003 |
| A0A8I5ZYK0 | Major urinary protein-like | -0.629 | 0.025 |
| A0A0H2UHF8 | Orosomucoid 1 | 2.686 | 0.002 |
| P20759 | Ig gamma-1 chain C region | 0.973 | 0.012 |
| A0A0G2K9Z3 | Nucleobindin 1 | -0.720 | 0.015 |
| A0A8I6ASQ4 | Prosaposin | -0.954 | 0.017 |
| A0A8I6AQI7 | Serpin family F member 2 | 0.796 | 0.038 |
| A0A8I6A4U0 | Ig-like domain-containing protein | 0.958 | 0.025 |
| A0A8J8XKZ6 | Complement C2 | 0.749 | 0.035 |
| P51886 | Lumican | -0.695 | 0.026 |
| F1M9B2 | Insulin-like growth factor binding protein 7 | -0.614 | 0.03 |
| A6JJ35 | Platelet-activating factor acetylhydrolase | -0.619 | 0.037 |
| A2RUV9 | Adipocyte enhancer-binding protein 1 | -0.604 | 0.027 |
| F1LS40 | Collagen type I alpha 2 chain | -0.779 | 0.008 |
| F8WG88 | Follistatin-like 1 | -0.596 | 0.04 |
| G3V6E8 | Myocilin | -1.673 | 0.002 |
| Q10758 | Keratin, type II cytoskeletal 8 | -2.991 | 0.018 |
| A0A0G2JST3 | Keratin, type II cytoskeletal 1 | -2.077 | 0.021 |
| Q9JLD2 | Neuroserpin | -1.031 | 0.029 |
| D4A6P1 | Seizure related 6 homolog like 2 | -1.033 | 0.009 |
| A0A8I5YCJ2 | Transcobalamin 2 | -0.605 | 0.033 |
| M0RBD5 | Ig-like domain-containing protein | 0.758 | 0.03 |
| A0A8I6APC0 | Immunoglobulin heavy constant epsilon | 1.401 | 0.018 |
| Q6IFV3 | Keratin, type I cytoskeletal 15 | -1.541 | 0.025 |
| Q6AXS4 | Renin receptor | -0.905 | 0.024 |
| Q6MG90 | Complement C4B (Chido blood group) | 0.603 | 0.043 |
| M0R838 | Ig-like domain-containing protein | 0.741 | 0.04 |
| G3V6E7 | Fibromodulin | -1.482 | 0.001 |
| A0A8I5ZDN9 | Complement C5 | 1.383 | 0.016 |
| D4ACA7 | receptor protein-tyrosine kinase | 0.803 | 0.037 |
| A0A8I6ACW2 | Ig-like domain-containing protein | 0.683 | 0.024 |
| A0A0G2K2V6 | Keratin 10 | -2.747 | 0.007 |
| F1LZ11 | Ig-like domain-containing protein | 2.035 | <0.001 |
| D4A8G5 | Transforming growth factor, beta induced | -0.649 | 0.02 |
| Q9Z0K6 | Scrapie-responsive protein 1 | -1.361 | 0.013 |
| P50430 | Arylsulfatase B | -0.924 | 0.012 |
| G3V826 | Transketolase | 0.935 | 0.011 |
| P55314 | Complement component C8 beta chain | 0.873 | 0.026 |
| B5DFC9 | Nidogen-2 | -0.943 | 0.012 |
| D3ZWD6 | Complement C8 alpha chain | 0.930 | 0.01 |
| A0A8I6ALJ7 | SPARC | -1.564 | 0.004 |
| A0A8I5ZLW9 | Ig-like domain-containing protein | 1.242 | 0.033 |
| A0A8I5ZZ90 | Tropomyosin 2 | 0.909 | 0.028 |
| A0A0G2JWX4 | Keratin 2 | -1.398 | 0.034 |
| Q7TMC3 | Hermansky-Pudlak syndrome 5 protein homolog | 1.561 | 0.008 |
| M0R6K0 | Laminin subunit beta 2 | -0.899 | 0.022 |
| Q62975 | Protein Z-dependent protease inhibitor | 1.335 | 0.014 |
| Q9EQP5 | Prolargin | -1.058 | 0.008 |
| Q63772 | Growth arrest-specific protein 6 | -0.865 | 0.036 |
| A0A0G2JTA6 | Insulin-like growth factor II | -1.013 | 0.021 |
| A0A8I6AA60 | Ig-like domain-containing protein | 0.860 | 0.011 |
| A0A8I6AKB2 | Profilin | 1.220 | 0.013 |
| Q5ZQU0 | Sushi, nidogen and EGF-like domain-containing protein 1 | -0.884 | 0.007 |
| Q5FWS5 | Lysyl oxidase homolog | -1.180 | 0.001 |
| A0A0A0MXV3 | Cell growth regulator with EF hand domain 1 | -1.208 | 0.007 |
| Q32Q07 | Leucine-rich repeat neuronal protein 1 | -1.105 | 0.042 |
| M0R7Q2 | Ig-like domain-containing protein | 1.219 | 0.014 |
| Q9WV75 | Spondin-2 | -0.843 | 0.011 |
| G3V7Y2 | Osteomodulin | -0.788 | 0.007 |
| A0A8I5ZYG7 | EGF containing fibulin extracellular matrix protein 2 | -0.659 | 0.039 |
| F1LMI3 | Cadherin 3 | -1.024 | 0.004 |
| Q9EQV6 | Tripeptidyl-peptidase 1 | -0.842 | 0.044 |
| P35446 | Spondin-1 | -1.239 | 0.007 |
| G3V6B1 | Transforming growth factor beta | -0.948 | 0.036 |
| A0A0H2UHH2 | Amyloid P component, serum | 1.679 | <0.001 |
| A0A0G2K828 | Ig-like domain-containing protein | 1.264 | 0.023 |
| Q7M0E3 | Destrin | 1.186 | 0.015 |
| Q7TPJ5 | 3'(2'), 5'-bisphosphate nucleotidase 2 | -0.947 | 0.03 |
| A0A0G2JXI9 | Histone H2B | 2.482 | 0.002 |
| P08494 | Matrix Gla protein | -1.171 | 0.002 |
| A0A8I5ZQS5 | Activated leukocyte cell adhesion molecule | -1.260 | 0.026 |
| P50115 | Protein S100-A8 | 4.681 | 0.005 |
| A0A8I6G6Z1 | Sortilin 1 | -1.742 | 0.023 |
| A0A8I6A289 | Histone H3 | 1.661 | 0.007 |
| A0A0G2K014 | Lymphocyte cytosolic protein 1 | 1.730 | 0.018 |
| A0A8I6AJ44 | Tenascin XB | -2.004 | 0.014 |
| A0A8L2QC84 | Coactosin-like F-actin binding protein 1 | 0.749 | 0.031 |
| F1M7F7 | Complement component C6 | 1.981 | 0.001 |
| P82995 | Heat shock protein HSP 90-alpha | 1.074 | 0.009 |
| Q5RKI0 | WD repeat-containing protein 1 | 1.040 | 0.011 |
| A0A0G2JWG1 | Alpha-mannosidase | -0.860 | 0.047 |
| P30349 | Leukotriene A-4 hydrolase | 1.446 | 0.006 |
| A0A8I6ABG6 | Talin 1 | 1.433 | 0.026 |
| A0A8I6A3A5 | Ig-like domain-containing protein | 1.988 | 0.01 |
| A0A8I6A7R6 | Ig-like domain-containing protein | 2.376 | 0.012 |
| A0A8I6AUI8 | Myosin light chain 6 | 1.287 | 0.006 |
| A0A8I6A1P8 | Rac family small GTPase 2 | 2.491 | 0.004 |
| A0A8I5ZLM3 | EF-hand calcium binding domain 14 | -0.952 | 0.03 |
| P05370 | Glucose-6-phosphate 1-dehydrogenase | 0.865 | 0.032 |
| A0A8J8XU90 | Myosin, heavy chain 9 | 2.246 | 0.001 |
| P68255 | 14-3-3 protein theta | 0.927 | 0.009 |
| A0A0G2K8V2 | Vinculin | 1.955 | 0.008 |
| P62804 | Histone H4 | 3.682 | 0.001 |
| A0A8I5ZV49 | Filamin A | 3.058 | 0.002 |
| Q91ZN1 | Coronin-1A | 2.063 | 0.006 |
| A0A8I5ZKF7 | Annexin | 3.878 | 0.017 |
| M0R7B4 | H1.3 linker histone, cluster member | 3.736 | <0.001 |
| Q9QXQ0 | Alpha-actinin-4 | 1.660 | 0.017 |
| F1LTN6 | Ig-like domain-containing protein | 0.846 | 0.049 |
| Q08163 | Adenylyl cyclase-associated protein 1 | 1.640 | 0.002 |
| A0A8L2QPP4 | F-actin-capping protein subunit alpha | 1.866 | 0.003 |
| A0A0G2K1A2 | Myeloperoxidase | 4.784 | 0.001 |
